# Climate Change and State of the Art of the Sustainable Dairy Farming: A Systematic Review

**DOI:** 10.3390/ani15202997

**Published:** 2025-10-16

**Authors:** Delane Ribas da Rosa, Nicole Costa Resende Ferreira, Carlos Eduardo Alves Oliveira, Alisson Neves Harmyans Moreira, Rafael Battisti, Derblai Casaroli, Matteo Barbari, Gianluca Bambi, Rafaella Resende Andrade

**Affiliations:** 1 Veterinary and Animal Science School, Federal University of Goiás, Goiânia 74690-900, GO, Brazil; rosa.delane@discente.ufg.br (D.R.d.R.); nicole.resende@yahoo.com.br (N.C.R.F.); 2 Academic Unit of Agricultural Engineering, Federal University of Campina Grande, Campina Grande 58429-900, PB, Brazil; carlos.alves@professor.ufcg.edu.br; 3 Agronomy School, Federal University of Goiás, Goiânia 74690-900, GO, Brazil; alisson.harmyans@ufg.br (A.N.H.M.); battisti@ufg.br (R.B.); derblai@ufg.br (D.C.); 4 Department of Agriculture, Food, Environment and Forestry, University of Florence, Via San Bonaventura 13, IT50145 Firenze, Italy; matteo.barbari@unifi.it (M.B.); gianluca.bambi@unifi.it (G.B.)

**Keywords:** dairy cow, global warming, heat stress, production sustainability, thermal indices, THI

## Abstract

**Simple Summary:**

Rising global temperatures driven by climate change pose a significant challenge for dairy farming. Heat stress represents a major constraint for dairy cattle, negatively affecting milk yield, feed intake, fertility, and overall animal welfare. This review examines recent studies on heat stress in dairy cows, with a particular focus on adaptive strategies employed within different production systems. The Temperature-Humidity Index (THI) emerged as the most commonly used indicator for assessing heat stress. The findings emphasize that strategies such as genetic selection, cooling technologies, optimized nutrition, and improved management practices are essential to mitigate the impacts of heat stress on dairy cows. While most research has been concentrated in the Northern Hemisphere, studies from South American countries remain limited, despite their prominence as major milk producers. A comprehensive understanding and application of effective adaptation strategies are critical to sustaining dairy production and safeguarding animal welfare under changing climatic conditions.

**Abstract:**

Climate change is causing an increase in global temperatures, with significant impacts on dairy production. This systematic review analyzes the challenges of new climate scenarios, focusing on the resilience and adaptation strategies of dairy systems. The PRISMA methodology guided the review process using the Scopus and Web of Science databases. After applying exclusion criteria, 30 articles published between 2015 and 2025 were selected. The studies included analyses of the effects of heat stress on animal parameters (I), socioeconomic parameters (II), and technological adaptation tools (III) in various geographic regions. Most research over the last decade addresses category (I), with a greater concentration in the Northern Hemisphere. The Temperature Humidity Index (THI) emerged as the main indicator of heat stress, associated with the physiological responses of dairy cattle. Heat stress reduces milk production, feed intake, reproductive performance, and overall animal health, often leading to increased slaughter rates. Adaptation strategies include genetic selection for heat tolerance, improved environmental monitoring, cooling technologies, and optimized nutritional and management practices, applicable to both pasture and feedlot systems. Despite being among the main producers, studies on the topic in South American countries are still scarce in the literature.

## 1. Introduction

The definition of sustainable animal production emerged from the widely disseminated concepts of animal welfare and climate change within the UN’s 2030 Agenda. In this context, the term “sustainable animal” recently arose, referring to an animal that is environmentally adapted, productive, efficient in converting feed into animal products (milk, meat, and wool), healthy, and yields low carbon footprints, all while ensuring its welfare [1].

However, climate change threatens animal production and food security by projected decrease in rainfall and increase in air temperature. Consequently, greater attention is needed across various factors related to livestock production efficiency to adapt to the present climatic realities [2]. These challenges are accompanied by significant socioeconomic impacts, also within the dairy sector [3], which is equally important as a source of income and nutrition [4]. Thus, heat stress in dairy farming can become an additional challenge for a world already concerned about future food security in the face of climate change.

It is important to highlight the dual role of dairy farms: they are both significant contributors to greenhouse gas (GHG) emissions and highly vulnerable to the effects of climate change [5]. Furthermore, under heat stress conditions, dairy cows exhibit reduced milk production efficiency, increased susceptibility to disease, and lower welfare, which in turn elevates their methane footprint [6,7].

Over the last decade, global milk production increased by 27% between 2013 and 2023, corresponding to an average annual growth rate of 2.6% [8]. India currently leads global milk production, followed by the United States, Pakistan, China, and Brazil. Beyond these major producers, countries in the Mediterranean region, the Middle East, the Indian subcontinent, the savanna regions of West Africa, the highlands of East Africa, Southeast Asia, and parts of South and Central America have also expanded their participation in the dairy market. However, in many developing regions, dairy sector growth remains constrained by factors such as poor feed quality, high disease prevalence, limited access to credit and technical training, and the low genetic potential of animals for milk production [9]. These disparities underscore critical knowledge gaps concerning the effects of climate change on the dairy sector in major milk-producing countries, which are essential to consider in the context of global production trends.

To address this global problem, it is highly relevant that studies on the animal environment consider possible future scenarios in order to adopt measures that minimize adverse climate impacts. Thus, there is a need to understand and develop adaptation strategies that include environmental management measures and animal health maintenance factors, including heat tolerance, to address the challenges arising from climatic conditions.

While several recent reviews have explored the effects of heat stress on dairy cows, a new approach is needed to jointly characterize productive parameters and thermal comfort, and the resulting socioeconomic and adaptive factors required to address emerging climate scenarios. Therefore, the objective of this literature review is to compile and critically analyze recent research on the impacts of climate change on dairy farming. We focus specifically on the resilience of production systems, present key tools and adaptation strategies, and identify critical research gaps on this globally relevant topic.

## 2. Materials and Methods

A Systematic Literature Review (SLR) was conducted to provide a comprehensive overview of the challenges faced by dairy cattle production systems due to climate changes, and recent efforts regarding adaptation measures. The “Preferred Reporting Items for Systematic Reviews and Meta-Analyses (PRISMA)” statement [10] was used as a guideline for the SLR due to its methodological rigor, aiming to minimize the possibility of arbitrary choices when selecting and incorporating studies from databases.

**Figure 1:**
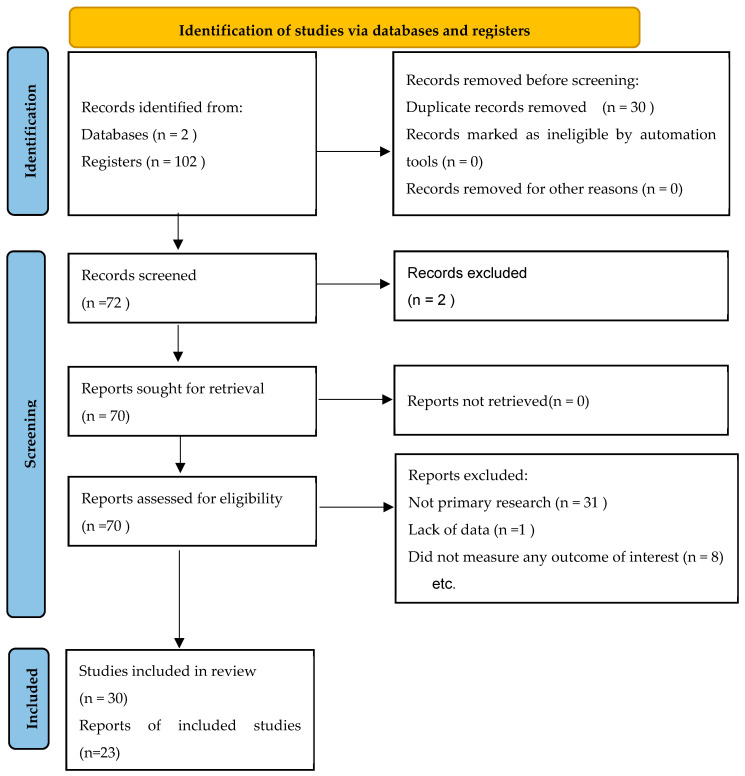
PRISMA 2020 flowchart of systematic reviews and research article selection methodology.

The research methodology comprised three main stages (Figure 1). First, the definition of the analysis field, establishing the fundamental concepts for the search. Second, the search string development and execution in the Scopus (ScP) and Web of Science (WoS) databases. Third, the extraction of information from the selected articles, followed by their analysis and discussion.

Based on the research objectives, keywords related to three key concepts were identified and used to define the search string in the ScP and WoS databases (Figure 1): (i) climate change and heat stress, (ii) welfare, animal behavior, physiological and productive variables, and (iii) adaptation strategies. Each section was then divided according to the study population, required intervention, comparison, and response variable of interest.

The keywords used in this search were: (“climate change” OR “global warming” OR “Climate impacts” OR “heat stress”) AND (“dairy farming” OR “dairy cattle” OR cow*) AND (“welfare” OR “well-being” OR “comfort” OR “nutrition” OR “physical environment” OR “health” OR “behaviour”) AND (“adaptation strategies” OR “mitigation strategies” OR “resilience”). The searches took place in May 2025, followed by data extraction in the following months.

### 2.1. Study Selection

To identify relevant articles, the search string was applied to the title, abstracts, and keywords fields indexed by the ScP database, and the ‘Topic’ search function in the WoS database. These databases collect the majority of scientific publications and are highly known for their extensive indexing, peer review, and multidisciplinary approach. As an initial filter, we chose to limit the search to the years 2015 to 2025, in English, Portuguese, and French. Subsequently, the Zotero^®^ version 7 reference manager was used to record, filter and delete duplicates from the research articles. A manual screening of articles was performed to avoid duplicates not detected by the software due to inconsistencies in titles. Authors DRdR (author 1) and RRA (author 9) performed this verification, followed by categorization performed by DRdR (author 1) and NCRF (author 2). Any disagreements during the selection process were resolved through discussion and consensus among all authors.

Non-primary research publications, such as literature reviews or similar, conference abstracts and book chapters were excluded. Similarly, articles that addressed species other than dairy cows, articles outside the context of production systems, and articles that addressed consumer research were not included in this study. Review articles were specifically excluded to avoid temporal bias, as their references often include primary research published outside the 2015–2025 timeframe.

Subsequently, only articles that addressed the following guiding questions were selected:

- Climate change and heat stress in dairy cows;
- Effects of heat stress on the physiology, performance, and behavior of dairy cows;
- Climate adaptation strategies in milk production systems.

### 2.2. Data Extraction and Processing

The articles included in this review were independently selected by two authors (DRdR and RRA). Data from the 30 eligible studies were carefully extracted and organized in an Excel^®^ spreadsheet. To ensure alignment with the objectives of this review, several aspects were thoroughly analyzed. Initially, the studies were categorized based on the specific objectives of each research into effects of heat stress (I), socioeconomic factors (II), and technological tools for mitigation and adaptation stemming (III) from climate change. For this purpose, studies were considered that aimed to (I) report any type of effect resulting from rising temperatures, (II) describe social realities or adaptations to climatic conditions, and (III) present technological tools for mitigating environmental effects or adapting to climatic conditions. Following this initial categorization, detailed information was extracted from each article, including the study’s geographic location, breeding system, cow breed, number of animals, their physiological stage, specific variables assessed, the thermal comfort index used, and other relevant parameters.

To facilitate data analysis and interpretation data were partially presented using graphic resources and visual representations. The graph provided by ScP was extracted to represent the temporal distribution of the publications found on the issue. Next, a word cloud was generated based on the metadata (titles, abstracts, and keywords) of the 30 articles selected after the complete screening process using the R software, Bibliometrix package, version 0.3.0. This approach helps identify the most frequently used terms within the selected literature.

The risk of bias was assessed using the Joanna Briggs Institute (JBI) checklist for analytical cross-sectional studies, consisting of eight items [11,12]. The analysis was conducted by two independent reviewers (DRdR and RRA), with any discrepancies resolved by consensus. The overall risk was classified as low when ≥70% of the applicable items were answered as “Yes”; high when <70% were “Yes” and “No” judgments corresponded to ≤50%; and unclear when “No” answers predominated (≥50% of applicable items or in cases of a tie).

Additionally, other articles were added to enhance the introduction and discussion; this strategy allowed the incorporation of a broader number of publications, favoring the development of a more robust and comprehensive conceptual base on the investigated topic. Additional scientific articles were added to supplement and substantiate the information and discussions on the topic. For the introduction and discussion of the results, 23 (Table A1) peer-reviewed articles on relevant topics were added.

## 3. Results

**Figure 2:**
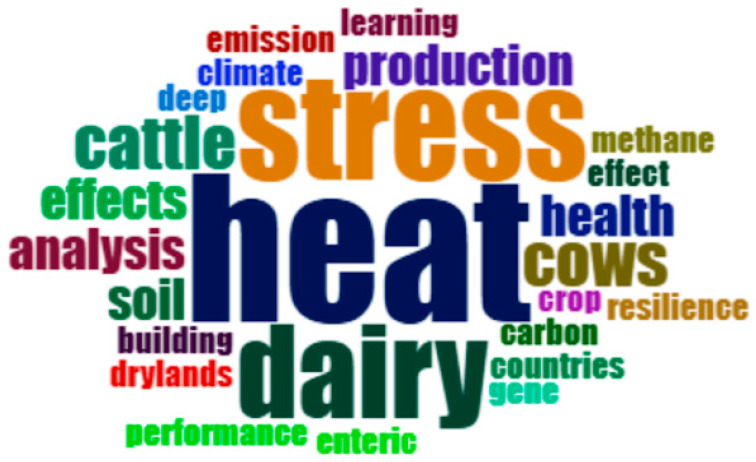
Word cloud generated from the 25 most frequently used words in the titles of articles included in the systematic review. Larger words indicate higher frequency of use.

A word cloud analysis was performed based on the selected articles (Figure 2). The most frequent terms identified in the abstracts were heat, stress, dairy, cow, production, and health. This distribution demonstrates that research has predominantly addressed the effects of heat stress on the health and productive performance of dairy cows, particularly in response to rising temperatures associated with climate change. The recurrence of these terms emphasizes the scientific relevance of the topic and delineates the primary concerns of the research community regarding dairy farming under global warming scenarios.

**Figure 3:**
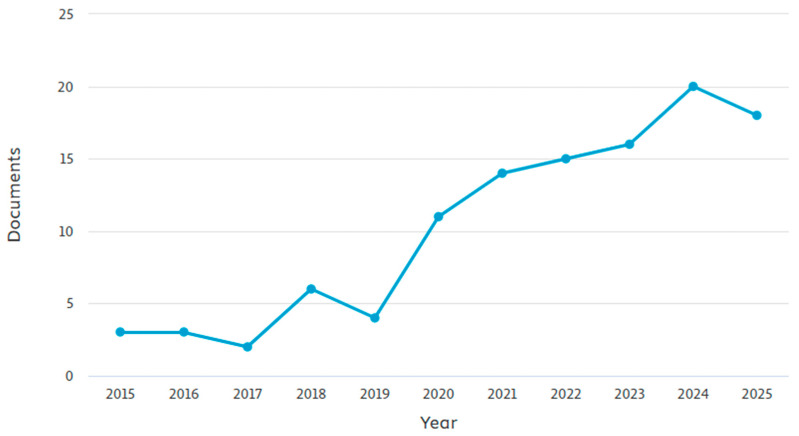
Articles temporal distribution from 2015 to 2025, based on Scopus searches.

The number of publications on this topic shows a significant upward trend, with a peak in 2024, which reinforces its growing global relevance (Figure 3). However, the geographic distribution of this research is notably uneven, with studies concentrated in specific regions of the world.

### 3.1. General Overview of Studies

**Figure 4:**
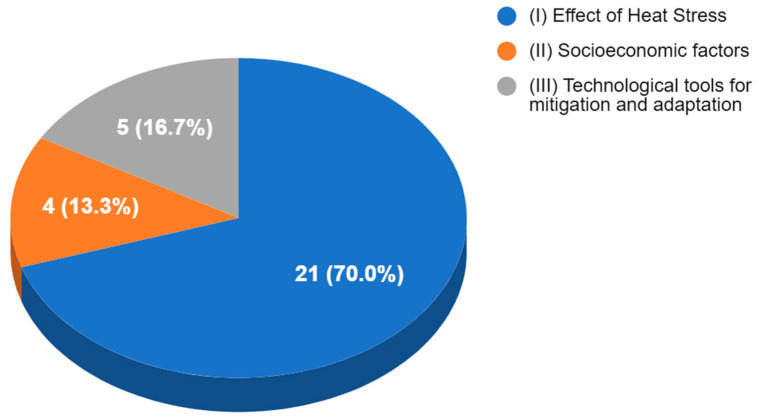
Categorical distribution of the 30 included articles.

The objectives and categorization of the 30 selected studies are summarized in Table 1. Our analysis revealed that the majority of articles (70%) were classified under Category I (Effects of Heat Stress). In contrast, 13.3% of studies focused on Category II (Socioeconomic Factors) and 16.7% on Category III (Technological Tools) (Figure 4). Although the majority of the literature on climate change is associated with the effects of the environment on animal performance, this compilation of scientific articles highlights a growing concern within the scientific community to investigate the human populations involved in production systems, recognizing their decisive influence on the sustainability of these systems.

The Northern Hemisphere contributed a larger volume of articles, accounting for 76% of publications compared to other regions worldwide, with 67% of these being experimentation with dairy cattle (category I). Countries in Asia hold the majority of publications, followed by Europe, North America, and Africa. In this exploratory approach, 23% of the total articles are from China, positioning it as the country that published the most on the topic in the last decade. All articles within category (III) were authored by researchers from China.

Southern Hemisphere countries had the lowest contribution to the topic, with only six experimental articles in category (I). Among these, Brazil was the most prominent contributor, publishing 6% of the total articles analyzed, which highlights a significant potential for increased research in the country.

Regarding category (II), two trials were conducted on the African continent, while two others were carried out in Asia.

### 3.2. Animals and Animal Thermal Comfort Indices

In the literature, various methods to evaluate heat stress in animals were identified, most commonly by correlating thermal comfort indices with the physiological responses of the animals. Among the thermal comfort indices, the Comprehensive Climate Index (CCI) is described by Arias et al. [16]. The CCI uses ambient temperature (°C), relative humidity (%), wind speed (m s^−1^), and solar radiation (W m^−2^) to classify the thermal environment as thermoneutral or stressful for Holstein cows. This index has been significantly employed to identify and characterize the thermal conditions of the environment at a given location.

The Temperature-Humidity Index (THI) is globally employed as a benchmark for measuring heat stress, as it quantifies the environmental thermal load an animal is exposed to, by integrating air temperature and relative humidity. However, the mathematical formula’s composition varies among different references. (Table 2). According to the articles compiled in this review, within category I we identified that 33% of the articles utilized the formula described by the NRC [42] to calculate the THI, although other authors were also cited (Table 2).

The range of THI exposure (minimum and maximum values) and the duration of the experimental period varied according to the specific THI formula used in each study (Table 2). Minimal variation was observed in the mean and minimum THI values across the references. However, it was surprising to note the extreme maximum THI values recorded in the experimental trials, which exposed the animals to thermal stress conditions as high as 90.

An important point to highlight is that the predominant production systems in the analyzed studies are intensive, primarily featuring Freestall facilities.

The CCI was reported in only one article (Table 2).

**Figure 5:**
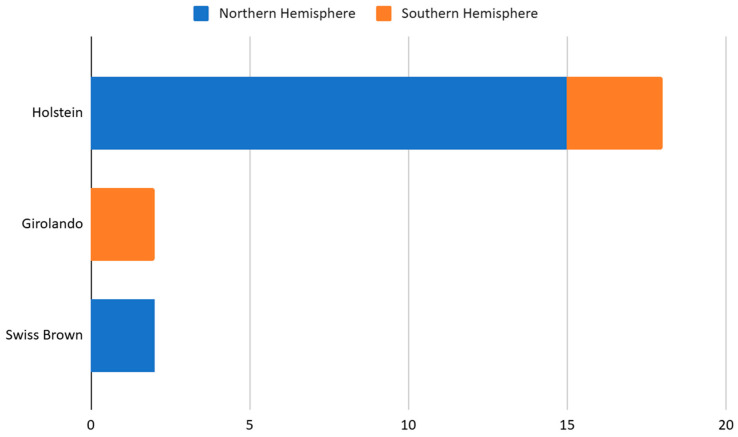
Geographical distribution of Category I studies according to breed.

The Holstein breed was the most studied in the selected Category I articles, particularly in the Northern Hemisphere. The extrapolation presented in Figure 5 of the number of articles in category I is due to the mutual study by Ceciliani et al. [19] with Holstein and Swiss Brown breeds. The two Girolando publications in the Southern Hemisphere were conducted in Brazil. Similarly, the two publications on the Brown Swiss breed were conducted in Italy.

### 3.3. Guiding Parameters of Heat Stress

**Figure 6:**
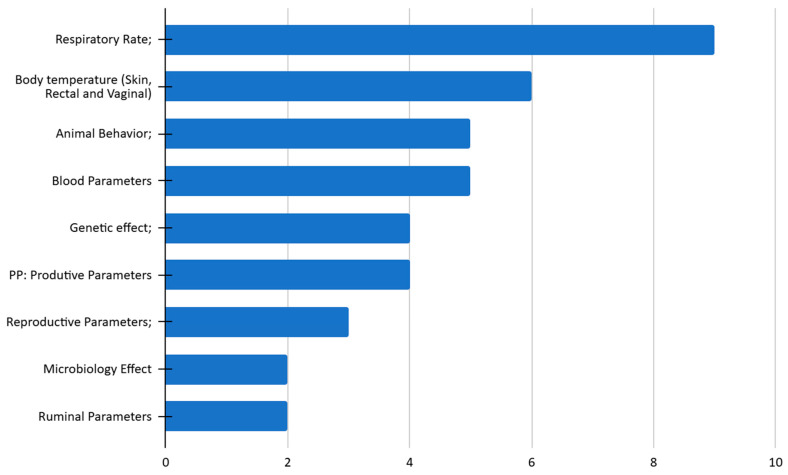
Variables in the 21 category I studies.

Among the articles classified as category I, 9 out of 21 used an elevated respiratory rate as the primary indicator of heat stress, followed by increased body temperature (6 articles), and changes in blood (5 articles) and behavioral parameters (5 articles) (Figure 6). The variables assessed in animals under heat stress were considered in combination, depending on the specific research objectives. Productive and reproductive parameters were evaluated in 4 and 3 studies, respectively, and reflect the impact of altered environmental conditions on the direct performance of dairy cows.

The JBI checklist items were defined as follows: Q1 assesses the clarity of inclusion and exclusion criteria for the study population; Q2 determines whether participants and the study setting are described in sufficient detail to permit replication and appraisal of external validity; Q3 evaluates whether the exposure or intervention was measured validly and reliably, including instruments and procedures; Q4 examines the use of a reference standard or recognized criteria for defining or diagnosing the condition, when applicable; Q5 considers whether potential confounders were explicitly identified; Q6 assesses whether appropriate strategies were implemented to control or adjust for these confounders (e.g., design features, matching, randomization, multivariable modeling); Q7 verifies that outcomes were measured validly and with reliability; and Q8 judges whether the statistical analysis was appropriate for the study design and data structure.

JBI appraisal showed strong reporting and measurement. Q1 (inclusion criteria) and Q2 (participants and setting) were clearly met in 28/30 studies (93.3%). Q7 (valid outcome measurement) and Q8 (appropriate statistical analysis) were also met in 28/30 studies (93.3%).

Exposure measurement (Q3) was valid in 25/30 studies (83.3%). Four studies were unclear. One study was not applicable due to simulation. Q4 (reference standard for the condition) was not applicable in 19/30 studies. It was met in 9/30 and unclear in 2/30.

Confounding remained the main limitation. Potential confounders were identified (Q5) in 18/30 studies (60.0%). Strategies to address confounding (Q6) were explicit in 13/30 studies (43.3%). Unclear reporting occurred in 11/30 for Q5 and 16/30 for Q6.

Overall methodological quality was moderate to high. Inclusion, setting description, outcome validity, and analytical adequacy were frequently met. Confounding identification and management were inconsistent. Future studies should pre-specify confounders, describe clear control strategies, report sample size and power, and standardize measurement and data flow. These steps will strengthen causal inference and comparability across studies.

## 4. Discussion

Our results allowed us to address the impacts of climate change on dairy production, aiming to make systems more efficient, with productive and healthy animals, effective feed management, and minimized greenhouse gas emissions. Data acquisition was somewhat limited due to the diversity of studies across different climatic conditions and production systems, coupled with a lack of detailed descriptions of experimental housing environments. This restricted a thorough analysis of optimal rearing conditions relative to specific regions or climates. Another factor that hindered this analysis was the inconsistencies in the application and citation of thermal comfort index formulas.

The effects of climate change can manifest in various ways, such as reduced rainfall and extreme events including droughts, storms, and floods [23]. Water scarcity, combined with limited pasture availability, negatively impacts milk production, increases disease incidence and mortality, and consequently affects the sustainability of dairy systems, particularly in tropical regions [13,25]. One way to mitigate these water-related problems is to improve water-use efficiency on farms by avoiding waste, reusing water within the system, and creating reservoirs for rainwater collection and storage.

Considering that much of the milk production in developing countries is pasture-based, agricultural practices that enhance soil water retention—such as no-till planting—combined with irrigation and fertilization using manure, represent short- to medium-term strategies.

Studies on the water footprint in dairy farming indicate that water use increases with rising temperatures. Accordingly, Le Riche et al. [46] highlight cooling systems as a measure to reduce the water footprint, alongside water reuse. Manure management for crop fertilization [47] is another strategy, with compost-bedded pack barns offering additional sustainability benefits, as the pre-composted bedding can be used as fertilizer after composting [48].

Agricultural credit systems and incentive programs play a fundamental role in guiding sustainable agricultural operations. Public policies should not only encourage practices that optimize water use but also foster strategies that mitigate the environmental impact of production. In this context, potential measures include benefiting producers who adopt water reuse technologies and waste management systems, as well as implementing programs aimed at enhancing product value and differentiation, aligning economic incentives with environmental and social sustainability goals.

### 4.1. Research Distribution and Socioeconomic Factors of Milk

This article compilation highlights the diverse realities within the global dairy sector, as reflected by the geographical distribution of research centers. While the largest milk-producing countries dominate scientific publications over the past decade, countries with high production potential, such as Brazil, contribute relatively little. This limitation arises because technologies validated under different conditions may not fully address local production challenges. This is particularly relevant for Brazil, as illustrated by Ferreira et al. [49], who found that climate change is projected to significantly increase heat stress (using THI) in the country’s primary dairy regions (South, Southeast, and Central-West) In contrast, countries like China have emerged as key players in the dairy market, and their strong representation in this review reflects the development of local experimental studies exploring technologies that support increased milk production.

Milk production capacity is directly influenced by socioeconomic factors, as well as the ability to sustain production systems under climate change. Key determinants for adopting measures to mitigate the effects of climate change and the environmental impact of production systems include education level and access to credit [17,23]. The presence of sociopolitical conflicts also plays a critical role in agricultural production [13]. Viable climate adaptation strategies for farmers include regular vaccination, the sale of animals with comorbidities, diversification of on-farm economic activities [17], and changes in management practices or cultivation of more resilient crop varieties [25].

Northern Hemisphere countries face greater challenges in maintaining pasture-based dairy systems due to climatic variations that cause pasture production instability, as reported by Gebrehiwot et al. [23] in Ethiopia and Koç et al. [25] in Turkey. In Australia, in the Southern Hemisphere, Osei-Amponsah et al. [37] highlight the difficulties of maintaining pasture-based dairy cows during summer, as animals reduce grazing time, increase water intake, and exhibit panting behavior, consequently decreasing milk yield. Similarly, studies on thermal comfort in pasture-based systems are widely discussed in Chile, such as the work by Arias et al. [16].

These findings emphasize that climate monitoring and forecasting are essential for implementing thermal comfort management strategies in pasture-based systems. Such strategies include natural or artificial shading [50] and sprinklers or fans in milking parlors [29]. Additionally, adjusting milking schedules can serve as an adaptation to high temperatures, prioritizing grazing during cooler hours around dawn and dusk, while scheduling milking during the warmer periods [51].

Despite this, there is a growing trend toward adopting confined dairy systems, such as free-stall barns and compost-bedded pack barns, driven primarily by their greater capacity to provide controlled and comfortable environments for milk production [52,53]. From an animal welfare perspective, confinement systems can mitigate unfavorable conditions, provide a good life for the animals [54], and reduce heat gain from solar radiation. Facilities with well-planned cooling systems can decrease the ambient temperature by 8.9 °C compared to the external temperature [29].

Management of confinement facilities has proven to be a key factor for achieving high productivity and herd longevity [6]. Leliveld et al. [27] observed variations in THI even among farms using the same confinement system. Strategies such as well-ventilated facilities, adjusting feeding schedules, avoiding handling animals during the hottest hours, and employing water spray systems combined with fans can help prevent drastic reductions in milk production during summer [14]. However, the implementation of cooling systems must be carefully planned according to region, location, investment, and electricity availability [37]. More efficient cooling systems may consume more energy compared to simple sprinklers and fans [38], highlighting the growing need for clean and sustainable energy production. Consequently, future research is required to develop technological innovations that enhance dairy production.

The great challenge is to ensure that sustainable innovations and strategies truly reach rural producers in an accessible and practical way, promoting their broad and effective adoption. Maintaining the three pillars of sustainability—economic, social, and environmental—becomes essential to enable a more efficient and climate-resilient dairy production chain that is environmentally responsible, ensures greater animal welfare, and achieves high long-term profitability. The consolidation of this path requires integrated public policies, investments in technical training, and incentives for the adoption of sustainable technologies, ensuring that dairy farming evolves in a way that is compatible with the Sustainable Development Goals (SDGs) and the demands of society.

### 4.2. Thermal Comfort Indices and Responses

The THI was the most widely used index in the literature, despite variations in its calculation formulas. Its main limitation is that it considers only temperature and humidity, ignoring factors such as solar radiation and wind, which are particularly relevant in pasture-based systems, as addressed by the CCI. Therefore, THI is better suited for confined and covered environments.

THI shows a strong correlation with productive and physiological parameters in dairy cows [18], as do other indices. Its measurement is closely linked to animals’ physiological and/or productive data, which is important because physiological changes precede declines in milk yield, serving as immediate benchmarks for thermal comfort monitoring and management adjustments, when necessary.

Although THI formulas combine the same climatic variables, the thresholds for the onset of heat stress may vary according to the reference used. For this reason, in this review the different THI applications were evaluated individually, according to their THI reference. In addition, the range between maximum and minimum THI values applied in each experimental trial may influence animal responses, particularly in fully enclosed environments such as climate chambers. For instance, Leliveld et al. [27] reported that THI variations from 51.8 to 85.2 reduced feed intake in Holstein cows.

At elevated temperatures, respiratory rate is the first physiological parameter to increase [22]. Prolonged panting leads to changes in blood composition, primarily an elevation in pH due to acid-base imbalance [15]. This physiological compensation for alkalosis results in increased bicarbonate excretion via urine, thereby reducing its circulating levels, which can cause ruminal acidosis [37]. In practice, the response to heat stress can be visually observed as an elevated respiratory rate or panting score, but continuous visual monitoring is laborious, time-consuming, and subjective [16,24]. Dairy cows with greater heat resistance tend to adjust their respiratory rate rapidly when facing thermal challenges [20].

Another parameter frequently used to assess heat stress is animal body temperatures. Rectal temperature was the second most common physiological reference parameter for heat stress, after respiratory rate. In a study by Corazzin et al. [21], the authors observed that cows subjected to a THI greater than 74 for 7 days showed a 0.7 °C increase in rectal temperature, a 1.8 kg/day reduction in milk production, and decreased consumption of forage (−6.9 kg DM) and concentrate (−2.4 kg DM). Additionally, Ceciliani et al. [19] found a 1.1 kg/day decrease in milk production for every degree that rectal temperature exceeded 38.2 °C in their studies.

Similarly, this was noted by Mendonça et al. [33] when exposing Girolando cows to a THI of 82. This phenomenon can be explained by Corazzin et al. [21], who associate the reduction in consumption with smaller meal sizes and not with feeding time. However, in the same study, the authors observed that when the cows are cooled the opposite is true; that is, the increase in feed intake is due to a longer feeding time. In grazing animals subjected to high temperatures, a change in grazing behavior and greater water intake are observed [37]. Decreased rumination activity is observed in cows exposed to heat [33]. The drop in rumination activity was described by Osei-Amponsah et al. [37] as being due to a lower intake of fibrous feed. Consequently, the effects of high air temperatures modify milk and microbiota composition, especially in Holstein cows [19].

In addition to consumption, the pathway food is degraded and digested also changes [15]. Gastrointestinal dynamics are directly related to feed utilization efficiency and methane emission, particularly at the ruminal level. In this context, a study by Park et al. [38] highlights the effects of heat stress on the ruminal microbiota. The authors found changes in the prokaryotic, fungal, and protozoan communities in Holstein cows subjected to extreme temperatures. These findings can be explained by the change in ruminal pH caused by blood alkalosis in cows with prolonged panting [37].

Kim et al. [24] report changes in the intestinal microbiota that decrease butyrate-producing bacteria, a short-chain fatty acid that plays a role in intestinal wall integrity. In ruminants, the modulation of the ruminal and intestinal microbiota is a determining factor for feed utilization efficiency.

Scientific literature consistently demonstrates that heat stress compromises milk production [14,33]. According to Chen et al. [20], heat acclimation is inversely related to milk production, meaning that cows that adapt more easily to prolonged heat conditions tend to have lower milk yields. Kim et al. [24] explain this reduced production as a diversion of glucose to meet the energetic needs of the immune system, particularly neutrophils [18]. Similar results were presented in the studies by Antanaitis et al. [15], showing lower glycemic levels in cows under heat stress. The low glycemic levels in these cows are explained by Mendonça et al. [33] as being due to a higher expression of cellular insulin receptors, leading to increased glucose uptake. In this context, projections by Chen et al. [29] indicate that animals subjected to heat stress can experience a reduction of up to 35% in milk yield.

Heat stress can lead to a drop of up to one-third in the insemination rate and conception risk [8]. In a study by Menta et al. [34], nulliparous cows exposed to a postpartum THI above 72 showed increased pregnancy losses. Cows exposed to high temperatures during the dry period have a 53.5% higher chance of retained placenta and a 24.5% higher chance of mortality [8]. Corroborating this, studies by Casarotto et al. [18] report that cows dried off 54 days before calving and subjected to an average THI of 77 have alterations in placental structures, which results in a shorter gestation period, lower calf birth weight, and reduced lactational performance of the dam. Nutrient restriction may be the placenta’s compensatory response to hyperthermia.

Studies by Menta et al. [34] demonstrated that reproductive problems resulting from high temperatures can decrease the chances of animals remaining in the herd by up to 60%. According to the authors, postpartum exposure to heat stress increases the chances of cow culling. The risk of mortality is higher, with the odds increasing by 1.1% for primiparous and 3% for multiparous cows, respectively. The risks of removal from the herd increase even more when cows are in prepartum thermoneutrality and are exposed to thermally stressful conditions postpartum, which increases the chances of developing metabolic diseases in early lactation.

### 4.3. Climate-Smart Agriculture Used to Increase Climate Resilience in Dairy Farming

Sensitivity to environmental changes may also vary with milk production level, parity [34], breed [19], and genetically predetermined thermoregulatory mechanisms [32]. Several studies have evaluated dairy cows’ tolerance to climate change or rising temperatures by monitoring their ability to maintain respiratory activity, body temperature, heart rate, and movement through automatic sensors. Animal-based indicators provide a more accurate assessment of heat stress due to variability among individuals, production systems, and farms [39]. For example, Li et al. [28] used automatic biosensors to measure heart and respiratory rates. Other sensors include vaginal implants and rectal thermometers, while thermography is used to measure surface body temperature [22,26,37].

Genetic selection for heat tolerance can exploit heritability and genotype–environment interactions, offering a promising approach to sustain productivity under high THI conditions, 76–90 [55]. The identification of genes linked to stress markers—such as rectal temperature, respiratory rate, and salivation score—provides a basis for selective breeding strategies [32]. Technological interventions, such as genetic improvements, nutritional interventions, and metagenomic applications, can help sustain milk production in changing climates [25,52] and reduce the negative effects on milk production and reproduction.

The use of locally adapted breeds, also known as native species, represents an alternative for sustaining livestock production, as these animals possess genetic adaptations to unfavorable conditions, such as high temperatures and the ability to digest lower-quality feed [35]. In contrast, choosing high-yielding dairy breeds often requires greater investment in infrastructure, since these animals increasingly depend on thermoregulatory mechanisms such as evaporative cooling [28]. Another alternative is to select genetically improved breeds with phenotypes that support body temperature regulation while maintaining significant productive potential within the tropical production system.

Holstein cows exhibit lower tolerance to temperature fluctuations than other dairy breeds [16,19,25]. Studies have shown that after four days of heat exposure, these animals become more susceptible to mastitis due to a weakened immune response and physicochemical changes in milk, leading to greater bacterial diversity compared with Brown Swiss cows [19]. Such changes are mainly reflected in reduced protein content and coagulation stability, findings also reported by Arias et al. [16]. However, in light of climate change, greater efforts will be necessary in diagnosing climate risks for timely decision-making to avoid losses that affect animal welfare conditions. Mathematical models can assist in directing actions in response to climate change (based on future scenarios) and in identifying animals that require greater attention [6,22].

Cresci et al. [22] developed a high-precision model capable of detecting cow responses based on their heat dissipation capacity. Recently, Chen et al. [6] used a predetermined model to simulate scenarios of productivity, health, and mortality in a herd of high-producing dairy cows, based on biological modifications. Liu et al. [29] conducted local projections relating the internal and external barn environment on a farm to THI and milk production. All models found negative effects on milk production associated with the estimated temperature increases.

The inclusion of plant-based bioactive compounds in diets can improve dry matter intake [56], protein metabolism, and immune regulation [57] in lactating cows subjected to heat stress compared to non-supplemented animals.

## 5. Conclusions

The main impacts of climate change on dairy cows are associated with exposure to high environmental temperatures, which negatively affect milk production, reproduction, and longevity. Records from different parts of the world highlight the magnitude of this issue for the sustainability of dairy systems.

This systematic review provides crucial insights into strategies for adapting to heat stress to ensure the health and welfare of dairy cows. The adoption of best management practices and properly designed facilities is essential for achieving more efficient and less polluting production systems. Similarly, adjustments in animal and forage management, shading, and supplementation can help sustain pasture-based systems under thermal stress.

Animal health must be continuously monitored, as it is directly linked to the economic viability of dairy farming. Only healthy animals can perform adequately, benefiting farms, farmers, and consumers alike. Dairy farming has been, is, and will remain a vital occupation, ensuring the production of agri-food goods necessary to feed the population while securing jobs across the sector.

Given the predominance of studies conducted in the Northern Hemisphere and the scarcity of research in South American countries, further regional investigations are recommended to assess the impacts of heat stress on dairy production under diverse tropical and subtropical edaphoclimatic conditions. This review also has the potential to inform public policies, particularly in education and credit, fostering the adoption of climate adaptation measures and supporting the persistence of dairy farming families in developing countries.

Additionally, there is a pressing need for regional and local research on new technologies—nutritional, genetic, and structural—tailored dairy production systems, which hold significant productive potential. Future studies could integrate local microclimatic analyses with physiological and productive data from dairy cows, combining monitoring technologies to develop regional predictive models to investigate vulnerability and resilience to thermal stress.

## Figures and Tables

**Table 1:** Summary of the objectives and categories of the 30 papers selected for this study.

Paper	Summary of Objectives	Thematic Axis *
[13]	To investigate dairy farmers’ adaptability to climate risks and their effects on dairy farm productivity and income.	(II)
[14]	To investigate the effects of HS on production and physiological parameters of Holstein cows.	(I)
[15]	To investigate and quantify the impact of heat stress on the milk fat-to-protein ratio (F/P) and the metabolic profile in dairy cows.	(I)
[16]	To assess the effect of the summer thermal environment on physiological responses, behavior, milk production and its composition on grazing dairy cows in a temperate climate region, according to the stage of lactation.	(I)
[17]	To improve our understanding of the effects of violent conflict on smallholder farmers’ adaptation to climate change impacts by comparing one mountain affected by sectarian conflict and one mountain affected by political instability.	(II)
[18]	To investigate the impact of heat stress, trials were conducted over the summer months of 2020, 2022, and 2023 in Florida.	(I)
[19]	To compare (1) the performance of 2 dairy breeds, namely Holstein and Brown Swiss, subjected to HS and (2) the different effects of HS on the milk microbiota of the 2 breeds in thermal comfort conditions and HS.	(I)
[20]	To model the impact of heat stress on dairy production and enteric CH4 emissions by aggregating its effects on milk production, reproduction, and health.	(III)
[6]	To investigate the effects of heat stress (HS) on blood, production, and physiological indicators in heat-tolerant and heat-sensitive cows.	(I)
[21]	To investigate the effect of heat stress and a cooling system on the feeding behavior of Italian Holstein Friesian dairy cows in late lactation.	(I)
[22]	To (i) develop an explicit model able to capture the dynamic phenomena and system delays that fit observed MY in dairy cows under HS, and (ii) present an initial attempt to estimate the system delays characterizing the cow response to HS needed to parameterize the model and discriminate among cow tolerant and not-tolerant to HS.	(I)
[23]	To investigate climate extremes and their associated effects encountered by smallholder livestock farmers in the Raya Alamata district of northern Ethiopia.	(II)
[24]	To understand the effects of heat stress on the health of dairy cows and observing biological changes.	(I)
[25]	To demonstrate how dairy farmers in the Thrace region are affected by climate change; the second is to investigate the adaptation methods they use to minimize farm-level negative effects and finally, to analyze the farm and farmer specific factors that determine the likelihood of adaptation.	(II)
[26]	To investigate G × E for heat tolerance in Brown Swiss cattle for several production traits (milk, fat, and protein yield in kilograms; fat, protein, and cheese yield in percentage) and 2 derivate traits (fat-corrected milk and energy-corrected milk).	(I)
[27]	To study the effect of hot weather conditions on known and novel behavioral parameters.	(I)
[28]	To investigate the application of a combined geothermal heat pump with a precision air supply (GHP-PAS) system for cooling dairy cows on a dairy farm.	(III)
[29]	To fill the research gap by thoroughly investigating heat stress levels and mitigation solutions in a cubicle dairy housing barn and a milking parlor.	(III)
[30]	To identify epigenetic differences between high and low immune responder cows in response to heat stress.	(I)
[31]	To investigate the genetic architecture of RT by estimating genetic parameters, performing genome-wide association studies, and biologically validating potential candidate genes identified to be related to RT in Holstein cattle.	(I)
[32]	To identify quantitative trait loci (QTL) regions associated with three physiological indicators of heat stress response in Holstein cattle.	(I)
[33]	To understand the relationship between milk yield, milk quality, and the expression of genes related to milk synthesis, cell apoptosis, and immune response in mammary cells of Girolando cows.	(I)
[34]	To determine the association of heat stress (HS) exposure during the periparturient period with production, health, reproduction, and survival during the first 90 d postpartum in dairy cows.	(I)
[35]	To investigate the effect of heat stress in milk yield of Girolando cattle in tropical climate and to identify the most appropriate statistical approach for evaluation and selection for heat tolerance in different breed compositions of Girolando.	(I)
[36]	To evaluate the lying postures of cows (lying with the head turned back (HB) and lying with the head up and still (HS)) as behavioral indicators of countermeasures.	(I)
[37]	To measure the impacts of summer heat events on physiological parameters (body temperature, respiratory rate and panting scores), grazing behavior and production parameters of lactating Holstein Friesian cows managed on an Automated Robotic Dairy during Australian summer.	(I)
[38]	To examine the direct effect of HS on the ruminal microbiota using lactating Holstein cows that were pair-fed and housed in environmental chambers.	(I)
[39]	To propose a deep learning-based model for recognizing cow behaviors and to determine critical thresholds for the onset of heat stress at the herd level.	(III)
[40]	To understand the genomic mechanisms of the 3 claw disorders dermatitis digitalis (DD), interdigital hyperplasia (HYP), and sole ulcer (SU).	(I)
[41]	To investigate a self-developed herbal formula as a dietary intervention to mitigate heat stress.	(III)

* (I) Effect of Heat Stress, (II) Socioeconomic factors, (III) Technological tools for mitigation and adaptation.

**Table 2:** Descriptive summary of the application of Thermal Comfort Indices in 21 studies assessing response variables to heat stress.

Reference Index	Mathematical Formula	Mean	Min.	Max.	AET (Day)
Kibler [43]	THI = 0.4 (DB + WB) + 15	76.5	68	85	60
Mader et al. [44]	CCI = AT + FRH + FWS + FSR	-	<20 °C	37.9 °C	16
NRC [42]	THI = (1.8 × AT + 32) − [(0.55 − 0.0055 × RH) × (1.8 × AT − 26.8)]	75	60	90	57
Thom [45]	THI = (0.8 × AT + (RH/100) × (AT − 14.4) + 46.4)	75.8	64.4	87.2	84

AET = Average exposure time (day), AT = Ambient Temperature (°C), RH = Relative Humidity (%), WB = Wet Bulb Temperature, DB = Dry Bulb Temperature, FRH = Relative Humidity Correction Factor, FWS = Wind Speed Correction Factor, FSR = Direct Solar Radiation Correction Factor. Maximum, mean, and minimum values were systematically extracted from the collection of studies included in this review.

## Data Availability

The data set is available on request to the corresponding author.

## Abbreviations

The following abbreviations are used in this manuscript:

CCI	Comprehensive Climate Index
ScP	Scopus
SDGs	Sustainable Development Goals
THI	Temperature Humility Index
WoS	Web of Science

## Appendix A

**Table A1:** List of added articles and their allocations in the text.

Author	Inclusion Local
[48]	Discussion
[57]	Discussion
[56]	Discussion
[7]	Introduction
[9]	Introduction
[49]	Discussion
[5]	Introduction
[55]	Discussion
[3]	Introduction
[4]	Introduction
[8]	Introduction
[47]	Discussion
[2]	Introduction
[51]	Discussion
[54]	Discussion
[11]	Methodology
[12]	Methodology
[52]	Discussion
[53]	Discussion
[10]	Methodology
[46]	Discussion
[50]	Discussion
[1]	Introduction
